# Clinical Outcome of Resected Non-Ampullary Duodenal Adenocarcinoma: A Single Center Experience

**DOI:** 10.3390/jcm12010210

**Published:** 2022-12-27

**Authors:** Soo Yeun Lim, Dong Il Chung, Hye Jeong Jeong, Hyun Jeong Jeon, So Jeong Yoon, Hongbeom Kim, In Woong Han, Jin Seok Heo, Sang Hyun Shin

**Affiliations:** 1 Division of Hepatobiliary-Pancreatic Surgery, Department of Surgery, Samsung Medical Center, Sungkyunkwan University School of Medicine, Seoul 06351, Republic of Korea; 2 Department of Medicine, Sungkyunkwan University School of Medicine, Suwon 16419, Republic of Korea

**Keywords:** duodenal cancer, non-ampullary duodenal cancer, clinical outcome

## Abstract

(1) Background: This study identified the clinical outcome and prognostic factors of resected non-ampullary duodenal adenocarcinoma (NADA) in a single tertiary cancer center. (2) Methods: The medical records of 109 patients with NADA who underwent curative surgery between 2000 and 2018 were reviewed retrospectively. (3) Results: The mean age was 62.4 years with a male predominance (70.6%). The majority of tumors were located at the 2nd portion (58.7%). Fifty-seven patients (52.3%) had symptoms at diagnosis. CA19-9 was elevated in 32 patients (29.4%). Of this cohort, most patients were diagnosed as stage III (64.2%). The median overall survival was 92.9 months, and the 1-, 3-, and 5-year survival rates were 84.4%, 71.6%, and 53.7%, respectively. In univariate and multivariate analysis, age, symptoms, CA19-9, and margin status were associated with overall survival and symptoms, CA19-9 and margin status were also associated with recurrence. When correlating symptoms with stages, patients with symptoms at diagnosis had more advanced stages (all *p* < 0.001). (4) Conclusion: Old age, elevated CA19-9, symptoms, and margin status were independent prognostic factors of NADA, and the patients with symptoms at diagnosis tend to have more advanced stages and a poor prognosis.

## 1. Introduction

Tumors arising in the non-ampullary parts of the duodenum are considered genuine duodenal cancer. These non-ampullary duodenal adenocarcinomas (NADA) are uncommon, representing less than 0.5% of gastrointestinal malignancies and constitute approximately 45% of small bowel adenocarcinomas (SBA) [1]. The analysis of NADA is frequently combined with other periampullary cancers originating from pancreatic, small bowel, and distal bile duct cancers, which leads to the absence of studies concerning the sole clinicopathologic characteristics and incidence of NADA [2]. In addition, various pathological factors such as lymph node metastasis, the positive resection margin, and cellular differentiation have been known to be prognostic factors; however, results are inconsistent throughout various studies likely due to small sample size, different standards, and misclassifications [3].

Recently, a study in Japan has observed a rising prevalence and a higher risk of NADA in Asia than in western countries, implying a high clinical relevance of this cancer as a potentially important area for research [4]. Approximately 40–60% of NADA are asymptomatic which leads to late detection. Therefore, duodenal cancers are detected at a far-advanced stage, making it the poorest prognosis among all small intestine cancers [5]. However, early detection through endoscopy and endoscopic mucosal resection of superficial non-ampullary duodenal epithelial tumor (early stage of NADA) has led to favorable outcomes indicating the importance of surveillance by esophagogastroduodenoscopies during routine checkups [6]. Identification of the risk factors associated with NADA, especially among South Koreans, will provide a framework for risk stratification that can be used in screening tests [7].

This single center retrospective study was conducted to determine the natural history of patients with operatively managed NADA. The study attempts to identify the clinical course and prognostic factors of NADA.

## 2. Materials and Methods

### 2.1. Study Design and Population

This retrospective study included patients who underwent surgery for NADA, with a curative intention, at the Samsung Medical Center (Seoul, South Korea) between January 2000 and December 2019. The inclusion criteria were defined as follows: (1) histologically diagnosed as adenocarcinoma by surgical biopsy; (2) tumor located at non-ampullary duodenum; and (3) no distant organ metastasis. This study protocol was approved by the Institutional Review Board of the Samsung Medical Center Sungkyunkwan University School of Medicine, Seoul, Korea, for clinical research [Registration No.: 2022-06-157-001]. This study was conducted in accordance with the ethical principles of the Declaration of Helsinki (1989).

Medical records including clinicopathologic, postoperative, and survival data were investigated retrospectively and outcomes of patients with survival and recurrence were also reviewed from the Samsung Medical Center Hepatobiliary-pancreas database in Seoul, South Korea. The following clinicopathologic parameters were collected: patient characteristics: gender, age, comorbidity (cardiovascular, pulmonary, and endocrine disease), presenting with symptoms at diagnosis, preoperative tumor markers (carcinoembryonic antigen [CEA], and carbohydrate antigen 19-9 [CA19-9]); pathologic findings: T stage, N stage, TNM stage according to the AJCC 8th edition, cellular differentiation, primary location, margin status, lympho-vascular invasion, perineural invasion; perioperative findings: operation name and adjuvant therapy; postoperative complication: a complication as defined by the Clavien–Dindo classification system [8], surgical site infection, intraabdominal abscess, pneumonia, acute kidney injury, biliary fistula, chylous ascites, postoperative pancreatic fistula (POPF), delayed gastric emptying (DGE), and postoperative hemorrhage. The follow-up duration was measured from the time of surgery until death or the last visit to the outpatient department.

### 2.2. Statistical Analysis

The student’s *t* test was used to compare continuous variables. The x^2^ test and Fisher’s exact were used to compare categorical variables. All continuous variables are summarized as median (SD) and all categorical variables are reported as frequencies (percentages). Overall survival (OS) and disease free survival (DFS) were estimated by the Kaplan–Meier method and the log-rank test was used for univariable survival analysis. Multivariable analysis of independent prognostic factors for OS and DFS was identified by using the Cox proportional hazard model. All tests were two-sided and a p-value under 0.1 was considered statistically significant. All statistical analyses were performed using the SPSS 27 version (IBM, New York, NY, USA).

## 3. Results

### 3.1. Demographic and Clinicopathologic Characteristics

**Figure 1:**
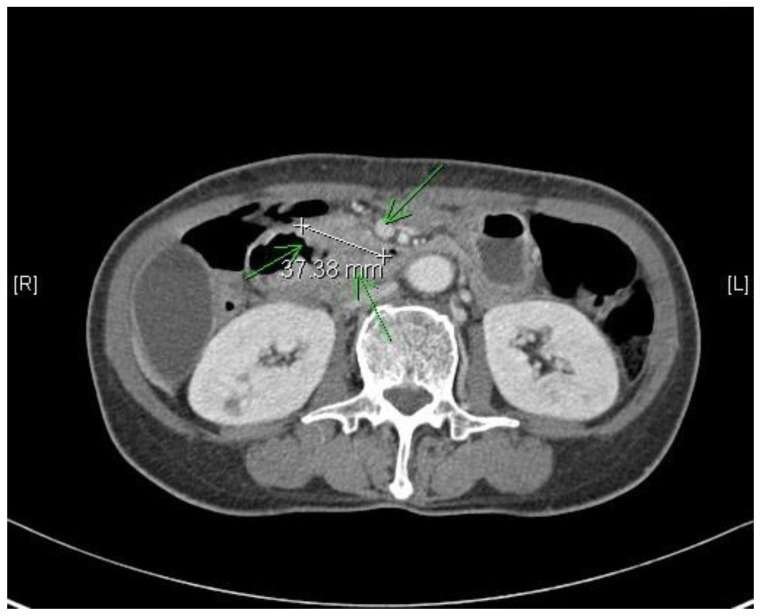
Illustration of non-ampullary duodenal adenocarcinoma. The green arrows indicate the duodenal adenocarcinoma from a 57 years old female patient, about 37 mm size tumor.

Table 1 and Table 2 summarize the demographic and clinicopathological characteristics of patients. Of the 109 patients, the mean age was 62.4 ± 10 years with a male predominance (70.6%). The presence of symptoms at diagnosis, found in 57 patients (52.3%), included: weight loss, jaundice, projectile vomiting, abdominal pain, melena, and hematochezia. CA19-9 was elevated in 32 patients (29.4%). The 2nd portion of the duodenum was the most common location of NADA (57.7%), followed by the 1st portion (11%). The mean tumor diameter was 3.7 ± 1.8 cm. Pancreatoduodenectomy (PD) or pylorus preserving pancreatoduodenectomy (PPPD) was performed in 96 patients (88.1%), duodenal segmental resection in five patients (4.6%), and a subtotal gastrectomy in seven patients (6.4%). Only one patient underwent a total pancreatectomy due to severe pancreatitis. The pathologic depth of the tumors was: T1 in 11 patients (10.1%), T2 in six patients (5.5%), T3 in 46 patients (42.2%), and T4 in 46 patients (42.2%). Seventy patients (69.7%) had lymph node metastasis. More than half of the patients were diagnosed with TNM stage III (64.2%). The majority of patients underwent R0 resection (94.5%) and 48 patients (44%) received adjuvant therapy following surgery. The Figure 1 shows illustration of duodenal adenocarcinoma.

### 3.2. Survival and Recurrence Pattern of NADA

**Figure 2:**
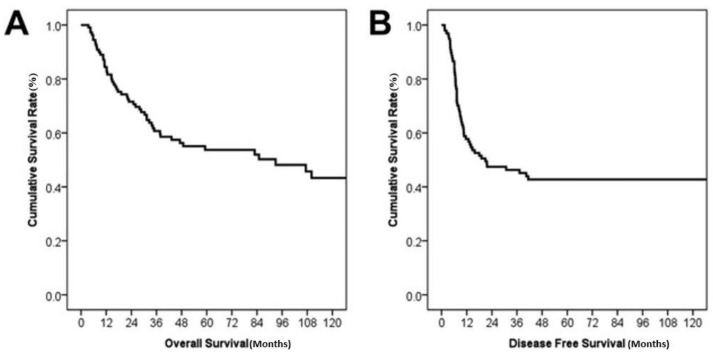
Kaplan–Meier survival curves of overall survival and disease free survival (*n* = 109). (**A**) The median overall survival was 92.9 months, and the estimated 1-, 2-, and 5-year survival rates were 84.4%, 71.6%, and 53.7%, respectively. (**B**) The median disease free survival was 20.9 months, and the estimated 1-, 2-, and 5-year disease free survival rates were 57.7%, 47.4%, and 42.7%, respectively.

**Figure 3:**
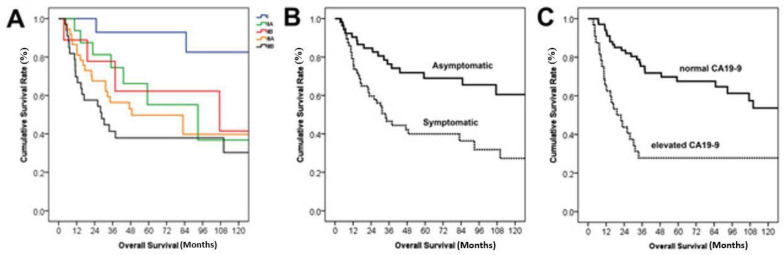
Kaplan–Meier survival curves of survival comparison according to TNM staging, symptoms, and levels of CA19-9. (**A**) In survival comparison according to the American Joint Committee of Cancer staging system, *p* values were 0.04 for I versus IIA, 0.009 for I versus IIIA, and 0.002 for I versus IIIB. The others were not statistically significant. (**B**) The median survival was not reached in asymptomatic patients (*n* = 52), and the median survival of symptomatic patients (*n* = 57) was 33.7 months (*p* = 0.001). (**C**) The median survival was not reached in patients with normal CA19-9, and the median survival of patients with elevated CA19-9 was 19.1 months (*p* < 0.001).

The survival and recurrence rates by Kaplan–Meier survival curves are shown in Figure 2. The median overall survival after surgery was 92.9 months. Overall survival rates at 1-, 2-, and 5-years were 84.4%, 71.6%, and 53.7% respectively. Recurrence developed in 55 patients (50.5%) during follow-up and the median DFS was 20.9 months. The estimated 1-, 2-, and 5-years DFS rates were 57.5%, 47.4%, and 42.7%, respectively. Figure 3 shows survival comparisons according to TNM stage, symptoms at diagnosis, and level of CA19-9. Stage I showed the best prognosis with statistical significance (*p =* 0.04 for I versus IIA, 0.009 for I versus IIIA, and 0.002 for I versus IIIB in Figure 3A). Patients with symptoms (Figure 3B) and elevated CA19-9 (Figure 3C) showed poorer survival (*p =* 0.001 and <0.001, respectively).

### 3.3. Uni- and Multivariate Analysis Affecting Survival

A multivariate analysis also showed that symptoms at diagnosis (hazard ratio [HR] 2.354, 95% confidence interval [CI] 1.236 to 4.486) and elevated CA19-9 (HR 2.821, 95% CI 1.555 to 5.117) were independent prognosis factors (Table 3). In addition, old age and margin status were other prognosis factors used in the multivariate analysis (HR 1.048, 95% CI 1.016 to 1.082 and HR 2.763, 95% CI 1.049 to 7.279, respectively). Moreover, a multivariate analysis for patients’ recurrence showed that symptoms at diagnosis in Table 4 (HR 3.720, 95% CI 1.807 to 7.657), elevated CA19-9 (HR 3.166, 95% CI 1.761 to 5.692), and Margin status (HR 3.447, 95% CI 1.174 to 10.124) were also independent prognostic factors.

### 3.4. Postoperative Outcomes

In terms of postoperative outcomes (Table 5), the median hospital stay was 15.9 days, ranging from 9 to 105 days. Major complications, with a Clavien–Dindo grade over 3, account for 11.0% of patients. Forty-seven patients had POPF (43.1%), 17 patients had a DGE (15.6%), and six patients had a postoperative hemorrhage (5.5%).

### 3.5. Relationship between Symptoms at Diagnosis and Tumor Stages

Table 6 shows the relationship between the presence of symptoms at diagnosis and tumor stages. The symptomatic group showed a higher T stage (*p <* 0.001) and N stage (*p <* 0.001) with statistical significance. Consequently, the TNM stage was also higher in the symptomatic group (*p <* 0.001).

## 4. Discussion

In this single center, retrospective study, we enrolled more than 100 duodenal adenocarcinoma patients that received curative surgery over the course of 20 years and investigated these cases in detail. Although there have been several large-scale multi-center studies involving data from the registered database, most previous studies suffer from selection bias as they choose not to distinguish between non-ampullary duodenal adenocarcinomas and periampullary cancers such as pancreatic, small bowel, and distal bile duct cancers [2]. Most non-ampullary duodenal cancer remains asymptomatic until a far-advanced stage, leading to its poor prognosis and high clinical relevance. Moreover, the fact that previous studies show that early detection through surveillance using esophagogastroduodenoscopies and endoscopic mucosal resection leads to favorable outcomes, implies the necessity of specified research [6,9,10,11]. We expect our single center study to have a lower risk of selection bias and our findings to likely reflect the sole clinicopathologic characteristics of non-ampullary duodenal adenocarcinomas and present the associated risk factors that may indicate the need for screening tests.

This study revealed that old age, CA19-9, and symptoms at diagnosis were prognostic factors for the survival of NADA after surgery. Previous retrospective studies reported various possible prognostic factors including CA19-9, gross appearance, tumor size, tumor invasion, lymph node metastases, TNM stage, lymphovascular invasion, perineural invasion, positive margin, poor differentiation, tumor markers (CEA and CA19-9), high lactate dehydrogenase, symptoms at diagnosis, and old age [10,11,12,13]. Our study results were similar to previous reports, however our findings also identified the tendency to have advanced T, N, and TNM stages when patients have symptoms at diagnosis, although these parameters were not found to be independent risk factors.

### 4.1. Incidence and Current Treatment of NADA

NADA, though it accounts for half of all SBA, showed a rate of 3.7 to 5.4 cases per 1 million persons per year, which is relatively rare [1]. The etiology of SBA including NADA is unknown. There are several theories, among which the theory that digestive contents cause irritation to the mucosa while passing through the intestine corresponds to both the facts that 2/3 of SBA occurs in duodenum and the SBA is rare compared to the large intestine [14]. NADA is a more common disease in men, accounting for a 2.4 times higher rate than females in this study. The difference in the ratio between men and women shows similar results to those of a study published in Japan, which showed a 1.7 times higher occurrence in males [4,10,14]. However, racial difference should also be considered as a study from a European population, little difference was observed between the male and female population and the occurrence of NADA. Regardless of race, the development of NADA was a common risk factor in the over 60s, at approximately 55%, and an increase in incidence with age was also observed in the multi-analysis results from this study. Hirashita et al. (2018) reported that 56% of tumors are most commonly located in the descending portion of the duodenum. This study showed a similar result, that 58.7% of the tumors were located in the 2nd portion of the duodenum [10]. The curative surgical procedures for NADA are various and depend on the tumor location. As shown in the previous results, the 2nd portion of the duodenum accounts for the largest proportion, therefore PPPD is the main surgical treatment [2,13]. The choice of surgical procedure for NADA occurring in the 3rd and 4th portion of the duodenum, is a controversial matter. Based on previous findings that lymph node metastasis and lymphovascular invasion are possible prognostic factors of disease recurrence and survival, some reports suggest that segmental resection could be an unsuitable treatment modality as it results in insufficient lymph node dissection compared to PPPD. Several studies found that for the early stage of NADA, local resection offered less morbidity, less mortality, and had a similar oncological benefit compared to PPPD [1,3,13,15,16]. From this study, it was not possible to make any conclusion regarding the links between significant survival benefits and tumor location, since only 15 patients had a tumor located in the 3rd and 4th portion. It is difficult to draw conclusions as to which operative procedure is the safest, most effective, and still provides an adequate surgical margin and minimal invasiveness. Therefore, further study is needed.

Even after surgery, there is a lack of a well-established adjuvant chemotherapy regimen and protocol. Haan et al. (2012) reported that SBA has a similar immunophenotypic pattern, molecular characteristics, and genome-wide DNA copy number aberrations with colorectal cancer. Consequently, many clinicians have applied the chemotherapy regimen used for colorectal cancer, to SBA, including NADA [2,17]. A combination of fluoropyrimidine and oxaliplatin (FOLFOX or CAPOX) is commonly utilized as an effective first line chemotherapy regimen for SBA, followed by an alternative front line combination of 5-FU and cisplatin or fluoropyrimidine and irinotecan (FLOFIRI) [2]. Adjuvant therapy, received by 44% of the total patients in this study was not a statistically significant factor for OS. This result was contradictory to the results of previous studies [2,5]. Due to its rarity, a large-scale investigation focusing on adjuvant therapy and its survival outcome will be a necessity in the future. As the lack of established principle of adjuvant therapy and prevention, periodic regular checkup is inevitably more emphasized as a prevention for NADA.

### 4.2. CA19-9 as a Prognostic Factor

Serum CA19-9 is a carbohydrate antigen expressed in tissue as a monosialoganglioside and a mucous protein. It is widely used as a biomarker for biliopancreatic malignancies, treatment response monitoring, and a marker of recurrence for pancreatic cancer. Elevated CA19-9 can be found not only in the detection of cancer, but also in a number of benign diseases including pancreatitis, cholestasis, acute hepatitis, chronic liver disease, diabetes mellitus, intestinal pulmonary disease, and even collagen vascular disease. Ventrucci et al. (2009) reported a possible mechanism for the elevation of serum CA19-9 with various benign diseases, especially those related to obstructive jaundice. In obstructive jaundice, CA19-9 can be overproduced by irritated bile duct cells and inflammatory proliferation of epithelial cells. Even accumulation of CA19-9 in the biliary tract can exacerbate the reflux of blood and bile into the circulation. Therefore, clearance of the biliary mucin decreases, making both the hepatic function and the ability to degrade the antigen decreased sequentially [9,18,19]. An elevated level of CA19-9 is associated with low survival of NADA, indicating that as the severity of the disease increases, NADA could cause obstructive jaundice by forming a mass effect that compresses the surrounding tissues.

### 4.3. Correlation with Symptoms and Tumor Stages in NADA

This study discovered that symptomatic patients had advanced stage NADA compared to non-symptomatic patients. Sakae et al. (2017) presented the common symptoms of small bowel cancer as stenosis-related such as abdominal pain or vomiting, and bleeding related such as melena or hematemesis [5]. The symptoms reported in this study are also consistent with these previous findings, including the presence of jaundice. Another study reported that early SBA patients rarely have stenosis-related symptoms because the products in the small intestine are mostly liquid [14,17]. We discovered that tumors with an advanced TNM stage result in the presentation of symptoms at diagnosis. Because many cases are asymptomatic until they reach the advanced stages of disease, early detection is delayed which results in a poorer disease prognosis. Therefore, this study emphasizes the importance of the early detection of asymptomatic NADA patients. A health checkup through esophagogastroduodenoscopy, which is already applied to adults over the age of 40, is a possible screening method, though there is a limit that the range of screening can only include up to 1st and 2nd portions of the duodenum. In addition, computerized tomography may be used as a screening method, however, considering the low incidence of disease and the cost values involved, discussion is inevitable regarding its use.

### 4.4. Limitation

Further limitations regarding this study include; first, this study is designed to be a retrospective study which has low statistical power. Second, almost half of the patients were presenting with symptoms at the first diagnosis. Additionally, the incidence of advanced TNM stage in patients was 64.2% of the total patients. Since the study was conducted at a large tertiary hospital, it is possible that the proportion of severely ill patients was high. Despite the several limitations, the strength of this study is that it confirmed the current clinical status for NADA, which is a rare but relatively common in Asia, and addressed the importance of early detection and appropriate surgical resection including the extent of lymph node dissection.

## 5. Conclusions

In conclusion, our study conducted a solitary data analysis of NADA clinical outcome and discovered that symptoms at diagnosis, aging, high CA19-9, and resection margin positive are independent prognostic factors for NADA. Patients with symptoms at diagnosis show an advanced TNM stage of NADA.

## Figures and Tables

**Table 1:** Demographic and clinical characteristics (*n* = 109).

Characteristics		N (%) or Mean (±SD)
Age		62.4 (±10.7)
Sex	Male	77 (70.6)
	Female	32 (29.4)
Comorbidity		
Cardiovascular	No	77 (70.6)
	Yes	32 (29.4)
Pulmonary	No	103 (94.5)
	Yes	6 (5.5)
Endocrine	No	90 (82.6)
	Yes	19 (17.4)
Symptom ^†^	Asymptomatic	52 (47.7)
	Symptomatic	57 (52.3)
Preoperative Lab		
CEA	Normal	80 (73.4)
	Elevated	12 (11.0)
	NA	17 (15.6)
CA19-9	Normal	67 (61.5)
	Elevated	32 (29.4)
	NA	10 (9.2)
Location	1st portion	12 (11.0)
	1st–2nd portion	9 (8.3)
	2nd portion	64 (58.7)
	2nd–3rd portion	9 (8.3)
	3rd portion	10 (9.2)
	3rd–4th portion	3 (2.8)
	4th portion	2 (1.8)
Tumor size		3.7 (±1.8)
Operation name	PD/PPPD	96 (88.1)
	Duodenum Segmental resection	5 (4.6)
	Subtotal gastrectomy	7 (6.4)
	Total pancreatectomy	1 (0.9)
Combined organ resection	No	93 (85.3)
	Yes	16 (14.7)
Adjuvant therapy	No	61 (56.0)
	Yes	48 (44.0)

^†^ Symptoms include jaundice, weight loss, abdominal pain, and projectile vomiting. SD, standard deviation; NA, not available; PD/PPPD, pancreaticoduodenectomy/pylorus-preserving pancreaticoduodenectomy.

**Table 2:** Pathological characteristics (*n* = 109).

Characteristics		N (%) or Mean (±SD)
T stage	T1a	3 (2.8)
	T1b	8 (7.3)
	T2	6 (5.5)
	T3	46 (42.2)
	T4	46 (42.2)
N stage	N0	39 (35.8)
	N1	37 (33.9)
	N2	33 (30.3)
TNM staging	I	14 (12.8)
	IIA	16 (14.7)
	IIB	9 (8.3)
	IIIA	37 (33.9)
	IIIB	33 (30.3)
Differentiation	Well	18 (16.5)
	Moderate	66 (60.6)
	Poor	25 (22.9)
Margin status	R0	103 (94.5)
	R1	6 (5.5)
Lymphovascular invasion	No	17 (15.6)
	Yes	30 (27.5)
	NA	62 (56.9)
Perineural invasion	No	18 (16.5)
	Yes	22 (20.2)
	NA	69 (63.3)

**Table 3:** Uni- and multivariate analysis identifying factors affecting patients’ survival (*n* = 109).

			Univariate Analysis		Multivariate Analysis	
Factors		Median Survival (mo)	Hazard Ratio	*p*	Hazard Ratio	*p*
Age			1.029 (1.002–1.058)	0.035	1.048 (1.016–1.082)	0.003
Sex	Male	107.3	Reference			
	Female	48.8	1.115 (0.620–2.006)	0.716		
Symptoms ^†^	Asymptomatic	Not reached	Reference		Reference	
	Symptomatic	33.7	2.577 (1.443–4.603)	0.001	2.354 (1.236–4.486)	0.009
CEA	Normal	92.9	Reference			
	Elevated	Not reached	0.979 (0.385–2.489)	0.965		
CA19-9	Normal	Not reached	Reference		Reference	
	Elevated	19.1	3.298 (1.850–5.879)	<0.001	2.821 (1.555–5.117)	0.001
TNM Stage	I	Not reached	Reference		Reference	
	IIA	92.9	3.984 (0.825–19.247)	0.085	3.003 (0.568–15.879)	0.196
	IIB	107.3	4.357 (0.797–23.823)	0.090	5.140 (0.902–29.284)	0.065
	IIIA	48.8	5.541 (1.283–23.934)	0.022	1.433 (0.259–7.918)	0.680
	IIIB	28.7	7.692 (1.798–32.905)	0.006	2.409 (0.467–12.440)	0.294
Tumor size			1.033 (0.894–1.194)	0.661		
Combined resection	No	107.3	Reference			
	Yes	31.3	1.623 (0.835–3.158)	0.153		
Location	1st–2nd portion	82.9	Reference			
	3rd–4th portion	92.9	0.842 (0.422–1.679)	0.625		
Margin status	R0	107.3	Reference		Reference	
	R1	7.0	3.966 (1.562–10.073)	0.004	2.763 (1.049–7.279)	0.040
Differentiation	Well	Not reached	Reference		Reference	
	Moderate	59.2	2.510 (0.978–6.440)	0.056	1.483 (0.404–5.436)	0.553
	Poor	34.9	2.679 (0.964–7.447)	0.059	2.825 (0.714–11.174)	0.139
Adjuvant therapy	No	85.0	Reference			
	Yes	Not reached	0.885 (0.508–1.541)	0.666		

^†^ Symptoms include jaundice, weight loss, abdominal pain, and projectile vomiting.

**Table 4:** Uni- and multivariate analysis identifying factors affecting patients’ recurrence (*n* = 109).

			Univariate Analysis		Multivariate Analysis	
Factors		Median DFS (mo)	Hazard Ratio	*p*	Hazard Ratio	*p*
Age			1.005 (0.979–1.031)	0.719		
Sex	Male	19.1	Reference			
	Female	20.9	0.906 (0.519–1.579)	0.727		
Symptoms ^†^	Asymptomatic	Not reached	Reference		Reference	
	Symptomatic	8.7	5.684 (2.972–10.870)	<0.001	3.720 (1.807–7.657)	<0.001
CEA	Normal	30.8	Reference			
	Elevated	6.2	1.152 (0.454–2.922)	0.965		
CA19-9	Normal	Not reached	Reference		Reference	
	Elevated	6.7	3.089 (1.764–5.410)	<0.001	3.166 (1.761–5.692)	<0.001
TNM Stage	I	Not reached	Reference			
	IIA	41.4	8.773 (1.096–70.200)	0.041		
	IIB	Not reached	3.955 (0.358–43.648)	0.262		
	IIIA	10.5	14.469 (1.950–107.357)	0.009		
	IIIB	8.9	19.378 (2.601–144.347)	0.004		
Tumor size			1.077 (0.941–1.233)	0.279		
Combined resection	No	20.9	Reference			
	Yes	18.0	1.174 (0.555–2.486)	0.674		
Location	1st–2nd portion	18.0	Reference			
	3rd–4th portion	41.4	0.724 (0.426–1.596)	0.566		
Margin status	R0	21.6	Reference		Reference	
	R1	4.2	4.003 (1.439–11.132)	0.008	3.447 (1.174–10.124)	0.024
Differentiation	Well	Not reached	Reference		Reference	
	Moderate	10.7	4.952 (1.523–16.102)	0.008	1.761 (0.482–6.442)	0.392
	Poor	14.0	5.376 (1.555–18.591)	0.008	2.264 (0.592–8.649)	0.232
Adjuvant therapy	No	Not reached	Reference			
	Yes	11.6	1.539 (0.900–2.632)	0.115		

^†^ Symptoms include jaundice, weight loss, abdominal pain, and projectile vomiting.

**Table 5:** Postoperative outcomes.

Factors	N (%) or Mean (±SD)
Hospital stay	15.9 (±12.4)
Complication ^†^	
Major complication ^‡^	12 (11.0)
Surgical site infection	2 (1.8)
Intraabdominal abscess	2 (1.8)
Pneumonia	2 (1.8)
Acute kidney injury	1 (0.9)
Biliary fistula	1 (0.9)
Chylous ascites	9 (8.3)
POPF	47 (43.1)
Delayed gastric emptying	17 (15.6)
Postoperative hemorrhage	6 (5.5)

^†^ Complication cases are duplicated. ^‡^ Major complication indicated Clavien–Dindo grade ≥ 3. SD, standard deviation; POPF, postoperative pancreatic fistula.

**Table 6:** Correlations between symptoms and tumor stages (*n* = 109).

Factors	Asymptomatic (%)	Symptomatic (%)	*p*
T stage			<0.001
T1a	3 (5.8)	0 (0.0)	
T1b	8 (15.4)	0 (0.0)	
T2	6 (11.5)	0 (0.0)	
T3	19 (36.5)	27 (47.4)	
T4	16 (30.8)	30 (52.6)	
N stage			<0.001
N0	34 (65.4)	5 (8.8)	
N1	8 (15.4)	29 (50.9)	
N2	10 (19.2)	23 (40.4)	
TNM stage			<0.001
I	14 (26.9)	0 (0.0)	
IIA	11 (21.2)	5 (8.8)	
IIB	9 (17.3)	0 (0.0)	
IIIA	8 (15.4)	29 (50.9)	
IIIB	10 (19.2)	23 (40.4)	

## Data Availability

Not applicable.
